# Towards a Better Molecular Diagnosis of *FMR1*-Related Disorders—A Multiyear Experience from a Reference Lab

**DOI:** 10.3390/genes7090059

**Published:** 2016-09-02

**Authors:** Sylwia Olimpia Rzońca, Monika Gos, Daniel Szopa, Danuta Sielska-Rotblum, Aleksandra Landowska, Agnieszka Szpecht-Potocka, Michał Milewski, Jolanta Czekajska, Anna Abramowicz, Ewa Obersztyn, Dorota Maciejko, Tadeusz Mazurczak, Jerzy Bal

**Affiliations:** 1 Department of Medical Genetics, Institute of Mother and Child, 17a Kasprzaka Street, 01-211 Warsaw, Poland; monika.gos@imid.med.pl (M.G.); daniel26a@wp.pl (D.S.); d.sielska-rotblum@czd.pl (D.S.-R.); aleksandra.landowska@imid.med.pl (A.L.); aspotocka@o2.pl (A.S.-P.); michal.milewski@imid.med.pl (M.M.); jolanta.czekajska@imid.med.pl (J.C.); anna.abramowicz@imid.med.pl (A.A.); ewa.obersztyn@imid.med.pl (E.O.); dorota.maciejko@wum.edu.pl (D.M.); t.mazurczak@wp.pl (T.M.); jerzy.bal@imid.med.pl (J.B.); 2 Department of Medical Genetics, Children’s Memorial Health Institute, 20 Al. Dzieci Polskich Street, 04-730 Warsaw, Poland; 3 Medgen, 27 Orzycka Street, 02-659 Warsaw, Poland; 4 Department of Immunology, Biochemistry and Nutrition, Medical University of Warsaw, 61 Żwirki Wigury Street, 02-091 Warsaw, Poland; 5 The Maria Grzegorzewska Academy of Special Education, 40 Szczęśliwicka Street, 02-353 Warsaw, Poland

**Keywords:** fragile X syndrome, FXTAS, FXPOI, *FMR1*, expansion, diagnostic, Southern blot, pre-screening PCR, TP-PCR

## Abstract

The article summarizes over 20 years of experience of a reference lab in fragile X mental retardation 1 gene (*FMR1*) molecular analysis in the molecular diagnosis of fragile X spectrum disorders. This includes fragile X syndrome (FXS), fragile X-associated primary ovarian insufficiency (FXPOI) and fragile X-associated tremor/ataxia syndrome (FXTAS), which are three different clinical conditions with the same molecular background. They are all associated with an expansion of CGG repeats in the 5′UTR of *FMR1* gene. Until 2016, the *FMR1* gene was tested in 9185 individuals with the pre-screening PCR, supplemented with Southern blot analysis and/or Triplet Repeat Primed PCR based method. This approach allowed us to confirm the diagnosis of FXS, FXPOI FXTAS in 636/9131 (6.96%), 4/43 (9.3%) and 3/11 (27.3%) of the studied cases, respectively. Moreover, the FXS carrier status was established in 389 individuals. The technical aspect of the molecular analysis is very important in diagnosis of FXS-related disorders. The new methods were subsequently implemented in our laboratory. This allowed the significance of the Southern blot technique to be decreased until its complete withdrawal. Our experience points out the necessity of implementation of the GeneScan based methods to simplify the testing procedure as well as to obtain more information for the patient, especially if TP-PCR based methods are used.

## 1. Introduction

The fragile X mental retardation gene (*FMR1*) is localized on chromosome X (Xq27.3). An expansion of the CGG repeat in 5′UTR region of the *FMR1* gene may cause three different clinical conditions: fragile X syndrome (number of CGG repeats over 200), fragile X-associated primary ovarian insufficiency (FXPOI) and fragile X-associated tremor/ataxia syndrome (FXTAS; in both number of CGG repeats within range 55–200).

Fragile X syndrome (FXS) is the most common inherited form of intellectual disability (ID), with a population prevalence of about 1/4000–9000 males and 1/7000–15,000 females [1,2,3]. Though the severity and clinical manifestation of the disease vary, FXS has several characteristic symptoms: intellectual impairment (mild to moderate), which may be accompanied by specific dysmorphic features like long face, large prominent ears, large jaw, and macroorchidism. In many cases, FXS is also considered a behavioral disorder as patients present with attention deficit hyperactivity disorder (ADHD) or autism spectrum disorder (ASD) [4].

The majority of FXS cases (>99%) are caused by the significant expansion of CGG trinucleotide repeats over 200, termed a “full mutation”, associated with methylation of the promoter and 5′UTR regions of the *FMR1* gene. The pronounced methylation leads to decrease of the expression of *FMR1* Protein (FMRP). In less than 1% of the FXS patients the molecular causes of the disease are, other than CGG expansion, mutations in *FMR1* gene. The deficiency of FMRP protein affects the synaptic plasticity in neurons and brain function and hence leads to the neurological manifestations observed in patients with FXS [5,6]. FXS is characterized by heterogeneous clinical penetrance. In almost all cases, men with the full mutation in the *FMR1* gene have more severe clinical symptoms as compared to women. The severity of the clinical symptoms might depend on the X chromosome inactivation pattern or the presence of a somatic mosaicism.

In the general population, the CGG repeats region of the *FMR1* gene is highly polymorphic. The normal size range of CGG repeats is lower than 44 [7] and these alleles are stably transmitted to the offspring. The alleles with 45–54 repeats are defined as the intermediate alleles (“grey zone” alleles) and are rather stable when passed to the progeny, and their expansion to a full mutation in the next generation has not been described. Nevertheless, about 16.9% of the intermediate alleles may expand to the premutation range when transmitted by the mother [8].

The number of CGG repeats in premutation alleles was estimated at 55 to 200 repeats. The premutation alleles are unstable and prone to expansion to full mutation (>200 CGG repeats) upon maternal transmission. The smallest allele that was described to expand to the full mutation in a single generation has 56 repeats [9]. The risk of the premutation expansion increases with the CGG repeat size and varies between 3.7% for the alleles with less than 59 CGG repeats and 100% for alleles with at least 99 CGG repeats [10].

The possibility of expansion depends not only on the premutation size, but also on the presence of the AGG interruptions in CGG sequence. The presence of the AGG sequence stabilizes the allele repeat number and correlates inversely with the risk of intergenerational transition to a full mutation. It has been observed that the AGG sequences are rarely present in the premutation alleles compared to in normal range alleles, which typically have two AGG interruptions [11].

Premutation carriers mostly have normal intellectual abilities, although some individuals have emotional, psychiatric and neurological problems [12]. The incidence of premutation alleles in the general population is estimated at 1/250–810 men and 1/130–256 women [13].

Recently, the premutation presence has been associated with other two diseases: fragile X-associated primary ovarian insufficiency (FXPOI) and fragile X-associated tremor/ataxia syndrome (FXTAS) [14,15]. Fragile X-associated primary ovarian insufficiency is defined as cessation of menses before the age 40 years. Approximately 20% of female carriers have FXPOI, although the rate varies depending on CGG repeat number. The greatest prevalence of FXPOI is between 70 to 100 CGG repeats [16].

Fragile X-associated tremor/ataxia syndrome is a neurodegenerative disorder in elderly people (>50 years) and affects mostly male carriers. The main clinical symptoms include tremors and cerebellar gait ataxia, Parkinsonism, neuropathy, and memory and executive function deficits followed by cognitive decline. FXTAS occurs in approximately 40% of men and 16% of women with the premutation in *FMR1* gene.

Since 1988, The Department of Medical Genetics has been a reference center for the molecular diagnosis of the FXS. Here, we summarize our experience with fragile X spectrum disorders molecular testing and present our results, which include data from 446 families with FXS, and 7 cases with FXTAS and FXPOI.

## 2. Materials and Methods

### 2.1. Patients

A total of 9185 individuals were tested, including probands, their relatives and prenatal cases. From this group, 2544 (27.7%) cases were patients of the Institute of Mother and Child, particularly of the Genetic Counselling Unit, Neurological Clinic or One-Day Outpatient Clinic. The remaining 6641 (72.3%) patients were referred for *FMR1* testing from other clinical centers in Poland. The patients with FXS suspicion or patients in whom FXS was considered in a differential diagnosis had a broad spectrum of the clinical symptoms: moderate to severe intellectual disability, developmental delay, speech delay, symptoms of autism spectrum disorders (ASD) and/or dysmorphic appearance.

The molecular analyses were performed according to the local ethical guidelines considering the genetic testing and the summary of the results was approved by the local ethical committee (Ethics Committee of the Institute of Mother and Child, Warsaw, Poland; number 15/2015, date of approval 18 June 2015).

### 2.2. Molecular Diagnosis

Molecular testing for FXS and *FMR1*-related disorders is based on the identification of the whole range of CGG expansion and also, in the case of the full mutation, the methylation status of *FMR1*. In our laboratory, the combination of two methods was used: pre-screening PCR (for female samples supplemented with Gene Scan method since 2009) and Southern blot hybridization and/or AmplideX *FMR1* PCR Kit (Asuragen, Austin, TX, USA) for samples with uninformative results from the primary analysis.

#### 2.2.1. Pre-Screening PCR

Genomic DNA was amplified using two primers: *FMR1* forward: 5′-GCTCAGCTCCGTTTCGGTTTCACTTCCGGT-3′, labeled with 6-FAM for GeneScan analysis and *FMR1* reverse: 5′-AGCCCCGCACTTCCACCACCAGCTCCTCCA-3′) [12]. The Expand Long Template PCR System (Roche Diagnostics, Hercules, CA, USA) and the reaction mixture containing 2.2 M betaine was used. The PCR cycling profile was as follows: denaturation at 98 °C for 10 min, 10 cycles at 97 °C for 35 s, 64 °C for 35 s, and 68 °C for 4 min; 25 cycles at 97 °C for 35 s, 64 °C for 35 s, 68 °C for 4 min with 20-s increment for each cycle; and a final extension at 68 °C for 10 min. The PCR fragments were subjected to electrophoresis on a 2% agarose gel and stained with ethidium bromide. The normal range alleles were expected to yield a PCR product of 331 bp (≈30 CGG repeats) to 501 bp (≈55 CGG repeats).

The fragment size analysis (Gene Scan), implemented to routine screening of all ambiguous female and male samples, allows for the identification of normal range alleles (including the grey zone) and small premutation alleles (up to 100 CGG repeats). The standard protocol for fragment analysis on ABIPrism 3130 sequencer (Life Technologies, Waltham, MA, USA) was used.

#### 2.2.2. Follow-up Analysis for Samples with Uninformative Results

For Southern blot hybridization, high molecular weight genomic DNA (10 μg) was digested with the *EcoRI* and methylation sensitive *NruI* restriction enzymes overnight. The digested DNA was separated in an agarose gel and then transferred to a nylon membrane in a semi-dry transfer. The Fragile X CHEMI™ DNA (Merck Millipore, Darmstadt, Germany) or Fragile X GLFXDig1 GeneProber™ Digoxigenin Labeled (Gene Link, Hawthorne, NY, USA) (Radiolabeled probes were used before the introduction of the chemiluminescent method and digoxigenin labeled probes) were used to Southern blot hybridization for the detection of specific DNA fragments according to the protocols suggested by the probe manufacturers. Detection was performed using a system dedicated to digoxigenin labelled probes (Sure Blot CHEMI Hybridization & Detection Kit, Merck Millipore, Darmstadt, Germany and Roche DIG Luminescence Detection kit, Roche Diagnostics, Hercules, CA, USA). Filters after hybridization were exposed to X-ray blue sensitivity film for at least 24 h or CCD camera for 2 h (ChemiDoc XRS+ Chemiluminescence System, Bio-Rad, Hercules, CA, USA).

Since 2014, we have implemented to our FXS testing protocol the Triplet Repeat Primed PCR-based method for premutation and mutation analysis, supplemented with MS-MLPA method (SALSA MLPA ME029 FMR1/AFF2 Kit) if requested. The AmplideX *FMR1* PCR Kit is used according to the manufacturer protocol.

### 2.3. X Chromosome Inactivation Analysis

The analysis of the X chromosome inactivation status in asymptomatic women with a full mutation in the *FMR1* gene was performed by the analysis of the highly polymorphic trinucleotide (CAG) repeats in the first exon of the human androgen-receptor gene (*AR*). Genomic DNA was digested with the methylation-sensitive restriction enzyme *HpaII* and amplified with primers according to the protocol described by Allen et al. [17]. The fragment size was analyzed using capillary electrophoresis. The area under the peaks corresponding to each band, from both a *HpaII*-digested (D) and undigested (UD) DNA, was determined. The degree of skewing was calculated as (D1/UD1)/(D1/UD1 + D2/UD2) when D1 and D2 represent the value of an area under the digested first and second peaks, UD1 and UD2 correspond to the area under undigested peak one and two [18]. A cutoff value for skewed X chromosome inactivation was set at 75%. To compare results in the target group, analysis of the control group was performed. We have tested *AR* locus inactivation in 35 females without clinical symptoms and a family history of neurogenetic disorders.

## 3. Results and Discussion

### 3.1. Testing the FMR1 Gene as a First-Line Test for Disturbances of Psychomotor Development

We have tested a total of 9185 individuals, including 7405 probands (6083 males, 1322 females) to confirm/exclude FXS as a cause of neurodevelopmental disturbances. In the tested patients for whom the clinical information was available, the most common symptoms were: intellectual disability (1882 patients), delayed psychomotor development (3873), autistic behavior (1448), delayed speech development (1270), dysmorphic features (1189) and/or hyperactivity/ADHD (1086).

The clinical diagnosis of FXS was confirmed by Southern blot hybridization and/or the TP-PCR test in 406 probands (Table 1). The full mutation was found in 385 males (4.19%) and 21 females (0.28%). If the mutation is detected in the patient, further analysis of the family members, especially symptomatic ones is indicated. Following cascade testing FXS diagnosis was confirmed in additional 119 individuals (Table 1). Together FXS was diagnosed in 525 cases out of 8034 (6.53%) studied symptomatic patients.

In the group of patients (1448) in whom autism or autism spectrum disorder (ASD) were diagnosed, the full mutation was found in 23 cases (1.58%). The overall frequency of co-occurrence of autism and FXS in the study group is 4.22% which is consisting with data reported in the literature [19].

In our laboratory, the FXS testing is performed for any type of delayed psychomotor or speech development, ID, ASD, including Asperger's syndrome, suspicion and/or in family members of affected patients [20]. Together in the group of 8596 individuals analyzed, we have identified 525 symptomatic individuals with a full mutation, resulting in the overall frequency 6.53%. The presented value is higher than the result published previously by our research group [21] and occurs in the range reported by other genetic centers (≈0.6%–15.3%) for the targeted populations [22]. In the patients, only 2792 (32.5%) had a diagnosis made entirely (clinical and molecular examination) in the Institute of Mother and Child. Nevertheless, given the prevalence in this population (6.36%), still the value is in the range reported in the other centers in the world.

Out of all patients with full mutation in the *FMR1* gene, in 111 cases (21.14%) somatic or methylation mosaicism was found. Somatic mosaicism (mutation together with premutation and/or normal alleles) was identified in 66 male and 24 female patients (90 cases, 17.14% of all mutation cases). The presence of both methylated and unmethylated (methylation mosaicism) alleles was found in 21 patients (4%), 18 of which were males. We have also identified 3 cases with both somatic and methylation mosaicism. This result is consistent with the published data where both types of mosaicism were present in approximately 12% and 6% cases, respectively. It is also known that individuals with somatic and methylation mosaicism have better intellectual/cognitive skills than patients with completely methylated expanded *FMR1* allele only and this was also obvious for our patients. The knowledge about the presence of mosaicism is especially important for proper genetic counselling and health care for FXS families [23,24].

The exclusion of the presence of the full mutation in affected individuals clinically suspected of FXS indicates the need for additional molecular analyses. Although the expansion of the CGG repeats and methylation in the *FMR1* gene is responsible for over 99% of FXS cases, in other cases, the FXS-like phenotype may still be caused by other mutations involving *FMR1 locus*. To date, as a result of the application of high throughput molecular techniques like array comparative genomic hybridization (aCGH) and massive parallel sequencing, 51 different mutations have been reported in the Human Gene Mutation Database [25]. These include large changes involving the whole or a part of the *FMR1* gene (33 gross deletions, 5 gross insertion/duplications, 1 complex rearrangement) and 12 point mutations (5 substitutions leading to missense/nonsense/splicing changes 6 mutations in regulatory sequences and 1 small deletion).

In our examined group, we have also reported premutation alleles in 26 patients (13 males and 13 females, 0.35%) with at least one of the following clinical symptoms: intellectual disability, autism, delayed speech development and microcephaly. According to the current state of the knowledge, the premutation in the *FMR1* gene, (EMQN guidelines) may be associated with developmental problems such as ASD or ADHD, intellectual disability or delayed psychomotor development [42]. However, in symptomatic patients with premutation the possibility of the presence of tissue mosaicism (full mutation present in tissues other than blood) should be considered [26]. In two cases with ID and premutation present in the blood samples, we had the possibility to also test DNA extracted from the patients’ fibroblasts. The presence of potential mosaicism was excluded in these cases and the premutation (59 and 56 CGG repeats) alleles were stably passed in three generations in patient families, suggesting that other mechanisms might be involved in the development of ID.

### 3.2. Testing for the FMR1 Gene in FXS Families

According to the applicable guidelines, the asymptomatic relatives in FXS families should be referred for the carrier testing. In our cohort, the premutation was identified in 329 individuals (306/840 females and 27/384 males) out of 1038 tested relatives (Table 1). In addition, the full mutation was found in 70 asymptomatic females, including 30 mothers of affected children. The absence of FXS features in these females can be explained by the non-random X chromosome inactivation as was reported by the others.

The skewed X inactivation is an uncommon observation, but according to the literature, is more prevalent in families with X-linked diseases [27]. Non-random X chromosome inactivation of the affected chromosome copy can prevent even mild disease symptoms in females. On the other hand, skewing of X-inactivation towards the unaffected chromosome copy can cause symptoms of X-linked disorders in females. For this reason, the analysis of the X chromosome inactivation in asymptomatic females with a full mutation may be helpful in explaining of the lack of FXS features in these women. Therefore, we have performed analysis of the X chromosome inactivation status in 16 families, in which an asymptomatic female was a carrier of the full mutation in *FMR1* gene.

Altogether, 52 females were tested, including 32 from the control group. Skewed X-chromosome inactivation (>75%) has been found in 10/20 carriers of the full mutation allele. In 3 cases the result was uninformative because of the homozygosity in the *AR locus*. In the control group, the frequency of non-random X inactivation was slightly lower (8/32). The median level of X chromosome inactivation was 74 and 68 in full mutation carriers and control females, respectively, which was statistically insignificant (*p* = 0.09, U-Mann Whitney non-parametric analysis). Therefore, our results do not support the hypothesis that the absence of the FXS clinical symptoms in women with a full mutation is due to the skewed X chromosome inactivation. We are aware of the limitations of our study as the status of X inactivation in blood cells might not reflect the molecular events in the neural cells, the studied group was quite small and the X inactivation status was tested only in one *locus*. The testing of other *loci*, e.g., direct examination of the CpG island methylation in the 5′UTR and promoter region, especially fragile X-related epigenetic element 2 (FREE2) of the *FMR1* gene was suggested as a more suitable method to analyze X chromosome inactivation in full mutation asymptomatic female carriers [28]. However, we believe that the observed differences in X chromosome inactivation should be more prominent if it is truly related to the degree of clinical presentation in females with a full mutation in the *FMR1* gene.

### 3.3. Prenatal Testing of the FMR1 Gene in FXS Families

Prenatal testing is one of the possibilities that should be offered to the FXS families with confirmed carrier status. As in the early stages of pregnancy (before 12 week gestation) the DNA methylation is not completed, the methylation of the CpG islands and promotor of the *FMR1* gene even in the presence of full mutation may not be established [29,30]. Thus, the results of the *FMR1 locus* analysis regarding methylation status in FXS prenatal cases may be unreliable. Therefore the testing of the material from amniotic fluid biopsy is recommended if the applied methods are based on the methylation analysis (e.g., hybridization or MS-MLPA) [26]. Until the implementation of the PCR-based methods for the *FMR1* expansion testing, the prenatal analysis was quite challenging due to the necessity of amniotic fluid cell culture and the need to extract high amounts of high-molecular weight DNA for the Southern blot hybridization.

In our laboratory, the majority of prenatal testing was performed on DNA extracted from cultured amniotic fluid cells (22/27). Only in four cases, the analysis was performed with DNA from chorionic villi in the beginning of the implementation of molecular methods to the FXS diagnosis. At this moment, the routine elements of the prenatal testing for FXS include: the fetus sex determination, CGG repeats analysis and maternal cell contamination [7].

We have performed 27 prenatal diagnoses for women—carriers of the *FMR1* premutation. An informative results were obtained for 22 fetuses. Prenatal testing allowed to identify the full mutation in 7 fetuses (3 male, 4 female), of which 2 had somatic mosaicism. In one case, the full mutation allele was identified together with the normal and premutation alleles, and in the other fetus concomitance of the mutation allele with premutation was found. In further 15 fetuses (10 male, 5 female), the full mutation and the diagnosis of FXS were excluded (Table 1). In four cases, the molecular testing was not finished because of problems with DNA yield and quality.

The interpretation of the results of the molecular prenatal testing for a FXS may be challenging especially in case of female fetuses with a full mutation in *FMR1* gene or fetuses with mosaicism. Up to 50% of women with the full mutation are affected, although females and mosaic cases have a less severe phenotype as compared to men with the full mutation. For this reason, the severity of the disease cannot be predicted prenatally despite of the identification of the presence of full mutation allele [31]. In three cases of planned prenatal FXS testing, the family agreed to stop the molecular analysis, once the fetus turned out to be female.

### 3.4. Analysis of the FMR1 Gene in FXTAS and FXPOI Patients

The presence of the premutation in *FMR1* is also associated with two other pathological conditions: FXPOI and FXTAS [32,33]. The testing in females with POI and elderly people with ataxia and tremor has been performed for three years, so the study group is quite small. Nevertheless, the premutation alleles were identified in 4/43 (9.3%) and 3/11 (27.27%) cases with clinical suspicion of FXPOI and FXTAS, respectively.

It was assumed that FXS-associated primary ovarian insufficiency affects around 20% of FXS carriers of the premutation allele. On the other hand, the premutation was identified in 0.8% to 7.5% of women with sporadic premature ovarian failure and in up to 13% of women with familial premature ovarian failure [31]. In our group, we have confirmed the clinical diagnosis of FXPOI in 9.3% of cases (Table 1), which is similar to the frequency described by others including one Polish research group (3 premutations per 39 examined POI cases, 7.9%) [34]. As the prevalence of premutation is quite high in patients with primary ovarian insufficiency, the molecular testing of the *FMR1* becomes a routine test in a diagnosis of primary infertility [35].

The first case of a late-onset neurodegenerative disorder related to *FMR1* gene was described in 2001. Since then, FXS family-based studies have shown that approximately 40% of male and 8%–16% of female premutation carriers, developed FXTAS [36]. In addition, the penetrance of the disease can vary depending on the age and number of the CGG repeats. The risk of FXTAS occurrence in male premutation carriers aged 50–59 is 17% and increases to 38%, 47% and 75% for men aged between 60–69 years, 70–79 years and over 80 years, respectively. The meta-analysis study revealed that 86% (19/22) of alleles identified in male patients with FXTAS are longer than 70 CGG (*p* < 0.001) as compared to approximately 22% of premutation alleles in the general population [37,31]. The occurrence of FXTAS is more common in male premutation carriers as compared to women [38]. However, in our group, we have identified one 54-year old woman referred from the Neurological Clinic with ataxia and tremor who was diagnosed with two premutation alleles with the same repeat number (exact number of 128 CGG repeats, the presence of two X chromosomes was confirmed by MLPA analysis with P095 Aneuploidy and ME029 FMR1/AFF2 Kits). Such an unusual result of *FMR1* testing, indicating the presence of two identical premutation alleles, might be due to uniparental disomy, but we did not have the possibility to test the patient parents.

In addition, several studies have shown that the frequency of the premutation presence in patients with adult-onset spinocerebellar ataxia is quite low (1.3%) [39]. In the Polish population, the incidence of FXTAS in a group of patients with this condition is even lower and was estimated at 0.56% [40]. With regard to these results, the testing for *FMR1* in spinocerebellar ataxia cases should be considered only if there are additional supporting signs (e.g., MCP sign and increased T2 signal intensity in the middle cerebellar peduncles) indicating the possibility of *FMR1* premutation involvement in the disease pathogenesis.

### 3.5. Effectiveness of Different Testing Protocols in FMR1 Gene Analysis

Before 2002, in our laboratory the suspected cases of FXS had been diagnosed by several methods: cytogenetic analysis of the fragile X site on the X chromosome 1980’s, RFLP (until 1993) or Southern blot genomic hybridization only (1993–2002). In 2002, the PCR pre-screening test was implemented in our laboratory and used in combination with Southern blot hybridization till mid-2014. Performing the PCR before of the Southern blot greatly facilitated the diagnostic process. During that time, the PCR method was sufficient to exclude FXS diagnosis or carrier status in 4314/5974 (72.2%) males and 617/1746 (35.3%) females referred to complete *FMR1* gene testing in our laboratory. One of the disadvantage of the pre-screening PCR method is that it cannot exclude the coexistence of a normal and an expanded allele (somatic mosaicism). Size mosaicism with normal alleles seems to be very rare, although possible. In our study only one male presented with a both premutation and full mutation alleles in addition to a normal allele, detected at very low levels.

In cases with uninformative results of the PCR analysis further Southern blot analysis was necessary (352 females and 445 males). In addition, the genomic hybridization was performed for 177 patients (114 females and 63 males) referred to our laboratory after an external pre-screening PCR test.

**Figure 1:**
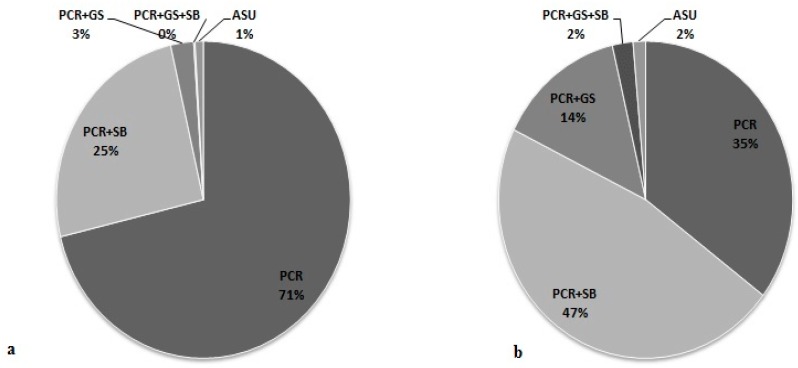
Percentage of molecular methods used to proper differantiation of the *FMR1* alleles. PCR—polymerase chain reaction, GS—Gene Scan, SB—Southern blot, ASU—Amplidex *FMR1* PCR kit, Asuragen. (**a**) Female; (**b**) Male.

In 2010, capillary electrophoresis (GeneScan method, GS) allowing the assessment of the number of CGG repeats was implemented for FXS diagnosis. The application of the GeneScan analysis not only allowed differentiation between normal, intermediate range and low premutation alleles, but also detection of normal alleles that differed by only one CGG repeat. This significantly reduced the number of additional Southern blot analyses, especially in the case of female testing. Besides that, the GeneScan method significantly reduced the cost and time required for the FXS molecular testing. Since the introduction of the GeneScan (GS) method, pre-screening PCR allowed an informative result to be established in 411/572 (71.8%) female patients as compared to 199/929 (21.4%) when this method was not a part of the diagnostic algorithm available in our laboratory. Moreover, in 43 individuals with intermediate alleles, the GS was adequate method to establish the informative result without the need of additional Southern blot testing that was further only used to detect high premutation alleles, full mutation and possible mosaicism (Figure 1a,b).

**Figure 2:**
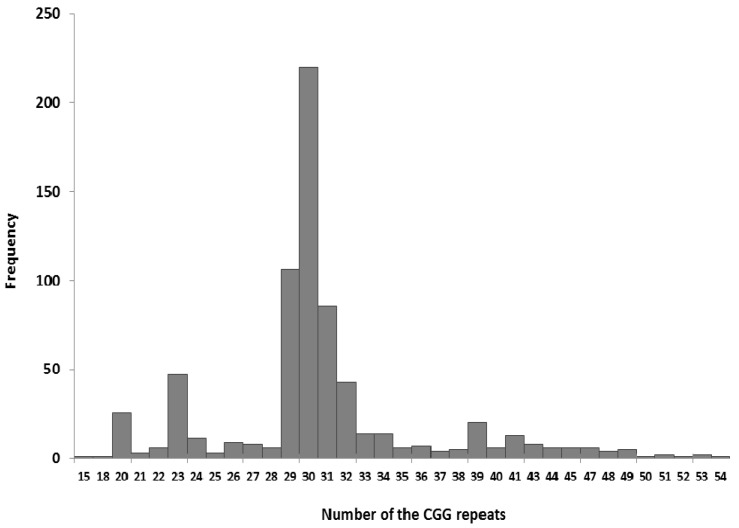
The size distribution of CGG alleles in *FMR1* gene. Histogram displays the frequency of alleles with different number of CGG repeats in normal range alleles, including intermediate alleles. The number of allele analyzed: 707, size distribution: 13–54 CGG repeats.

On the basis of the GeneScan results, the mean number of CGG repeats for the examined population was estimated at 30.7 ± 5.8 repeats. The most frequent allele in the Polish population has 30 CGG repeats and a frequency of 0.31 (220/707; Figure 2).

Recently, we have implemented, in the routine diagnostics, the Triplet Primed PCR based assay and AmplideX *FMR1* PCR Kit (Asuragen) [41]. So far, using this approach we have diagnosed 256 patients (82 male, 174 female), mostly individuals from FXS families. In this group, we have confirmed FXS in 74 cases (27.7%) and FXS carrier status in 52 (19.6%). When compared to the Southern blot analysis, testing with the Asuragen method is less time-consuming and seems to be more cost-effective. It also needs less input of the material. On the other hand, this method does not allow evaluation of the methylation status. To do this, additional analysis is required, such as MS-MLPA (male) and/or Amplidex mPCR (male and female). The analysis of the methylation is particularly necessary for patients with suspicion of the FXS and carrying only premutation. According to the current state of knowledge, the premutation can be partially methylated and cause mild expression of the FXS phenotype [26,42].

Asuragen TP-PCR method allows more effective detection of somatic mosaicism. In our group of patients, premutation and mutation alleles were co-identified in 24 cases (32.4%, 16 male, 8 female) with TP-PCR method, which represents 21.6% (24/111) of all identified mosaics. We have also used this test in prenatal diagnosis and were able to obtain the results for the DNA extracted from non-cultured amniotic fluid cells. Despite the high sensitivity of this method, routine prenatal diagnosis is performed on the DNA isolated from cultured amniocytes. Until now, four prenatal diagnosis were performed using TP-PCR based method. In two cases analysis revealed the presence of full mutation (in one male and one female fetuses), also one premutation (male fetus) and one normal allele were detected (male fetuses).

**Figure 3:**
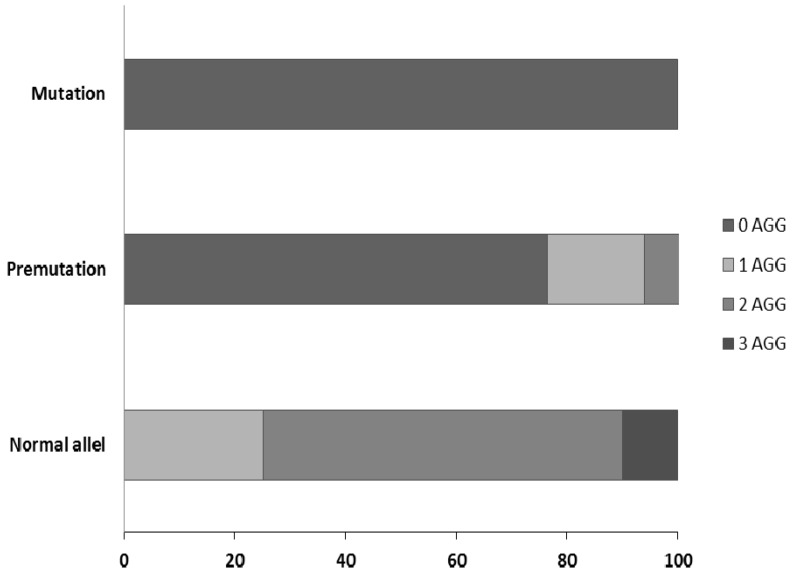
Number of the AGG interruptions (in percentage) in full mutation, premutation and normal alleles in a group of patients analyzed by TP-PCR (Asuragen).

The main advantage of the TP-PCR is the ability to determine the exact number of CGG repeats up to 200. In addition, this method makes it possible to estimate the number of AGG interruptions. Among 51 patients with confirmed presence of the premutation allele, the analysis has shown a high variability in the number of CGG repeats (range: 57–186, median: 88). In this group, 39 (76.5%) of carriers have no AGG sequence, and 9 (17.6%) and 3 (7.8%) have one or two AGG interruptions, respectively. In patients with the full mutation, no AGG interruption was present. In contrast, no allele without AGG was identified in people with *FMR1* alleles from the normal range (Figure 3).

These data is consistent with the literature and demonstrate that AGG interruptions occur less frequently in expanded alleles of the *FMR1* gene [43]. Information on CGG repeats allele size and on the number of AGG interruptions in carriers, is very helpful in genetic counseling in families with FXS.

Nevertheless, the method offered by Asuragen has some limitations. It does not allow assessment of the methylation status of the *FMR1* gene, but this disadvantage is also present for other PCR-based methods offered by other companies (e.g., Abbott—FMR1 TP-PCR and Sizing PCR, Perkin-Elmer—FragilEase™ PCR assay, Biofactory—FastFraX FMR1 Identification Kit and FastFraX FMR1 Sizing Kit). Therefore, the use of additional kits to define methylation status (Asuragen—Amplidex *FMR1* mPCR, Biofactory—FastFraX FMR1 Methylation Status Kit, MRC-Holland—SALSA MLPA ME029 FMR1/AFF2) should be considered, although this increases the analysis cost.

## 4. Conclusions

Molecular testing for FXS is one of the primary tests performed in patients with intellectual disability and delayed psychomotor development. As accessibility to genetic counselling and social awareness of the genetic basis of the diseases increase, there will be a need for the development of rapid and reliable methods for molecular testing. The application of PCR-based methods during the last several years greatly accelerated the process of FXS testing and significantly lowered the age of the diagnosis of the FXS in Polish patients. Our over 20 years’ experience as a reference laboratory clearly indicate that the application of new molecular methods in *FMR1* gene analysis greatly improves the effectiveness and decreases the time consumption of FXS diagnosis.

## Figures and Tables

**Table 1:** Summary of the results of molecular diagnosis of the FXS in the Polish population.

	Full Mutation	Premutation	Normal	Total
	*N* = 636	*N* = 389	*N* = 8160	*N* = 9185
**FXS/ID probands**	**406**	**30**	**6969**	**7405**
Males	385	18	5680	6083
Females	21	12	1289	1322
**Symptomatic relatives testing**	**119**	**19**	**491**	**629**
Males	102	1	330	337
Females	17	8	161	192
Brothers	73	3	201	70
Sisters	9	0	57	68
Mothers	1	4	44	65
Other relatives	36	3	189	334
**Asymptomatic carrier testing**	**104**	**333**	**638**	**1075**
Males	0	27	226	384
Females	104	306	412	840
Mothers	38	229	215	449
Sisters	47	13	97	141
Brothers	0	3	89	92
Fathers	0	2	17	19
Grandfathers	0	14	7	22
Aunts	5	40	28	73
Other relatives	14	33	185	242
**Prenatal diagnosis**	**7**	**0**	**15**	22 *
	2—somatic mosaicism			
Male	3	0	10	13
Female	4	0	5	9
**FXTAS**	**0**	**3**	**8**	**11**
**FXPOI**	**0**	**4**	**39**	**43**

* The data in the table includes only prenatal cases in which the informative results were obtained.

## References

[1] Crawford D.C., Acuña J.M., Sherman S.L. (2001). FMR1 and the fragile X syndrome: Human genome epidemiology review. Genet. Med..

[2] Hirst M.C., Knight S.J., Christodoulou Z., Grewal P.K., Fryns J.P., Davies K.E. (1993). Origins of the fragile X syndrome mutation. J. Med. Genet..

[3] Coffee B., Keith K., Albizua I., Malone T., Mowrey J., Sherman S.L., Warren S.T. (2009). Incidence of Fragile X syndrome by newborn screening for methylated FMR1 DNA. Am. J. Hum. Genet..

[4] Bailey D.B., Raspa M., Olmsted M., Holiday D.B. (2008). Co-occurring conditions associated with FMR1 gene variations: Findings from a national parent survey. Am. J. Med. Genet. A.

[5] Bagni C., Klann E., Powell C.M., Monteggia L.M. (2013). Molecular functions of the mammalian fragile X mental retardation protein: Insights into mental retardation and synaptic plasticity. The Autisms.

[6] Rzonca S.O., Gos M.E. (2012). Rola białka FMRP w prawidłowym funkcjonowaniu organizmu oraz patogenezie zespołu łamliwego chromosomu X. Postęp. Biol. Komórki.

[7] Biancalana V., Steinbach P., Glaeser D. (2013). EMQN best Practice Guidelines for molecular analysis and reporting in Fragile X mental retardation syndrome (FXS), Fragile X-asociated tremor/ataxia syndrome (FXTAS), and Fragile X-associated primary ovarian insufficiency (FXPOI). Eur. Mol. Genet. Qual. Netw..

[8] Nolin S.L., Glicksman A., Ding X., Ersalesi N., Brown W.T., Sherman S.L., Dobkin C. (2011). Fragile X analysis of 1112 prenatal samples from 1991 to 2010. Prenat. Diagn..

[9] Fernandez-Carvajal I., Posadas B.L., Pan R., Raske C., Hagerman P.J., Tassone F. (2009). Expansion of an FMR1 grey-zone allele to a full mutation in two generations. J. Mol. Diagn..

[10] Nolin S.L., Brown W.T., Glicksman A., Houck G.E., Gargano A.D., Sullivan A., Biancalana V., Bröndum-Nielsen K., Hjalgrim H., Holinski-Feder E. (2003). Expansion of the fragile X CGG repeat in females with premutation or intermediate alleles. Am. J. Hum. Genet..

[11] Yrigollen C.M., Durbin-Johnson B., Gane L., Nelson D.L., Hagerman R., Hagerman P.J., Tassone F. (2012). AGG interruptions within the maternal FMR1 gene reduce the risk of offspring with fragile X syndrome. Genet. Med..

[12] Loesch D.Z., Bui M.Q., Hammersley E., Schneider A., Storey E., Stimpson P., Burgess T., Francis D., Slater H., Tassone F. (2015). Psychological status in female carriers of premutation FMR1 allele showing a complex relationship with the size of CGG expansion. Clin. Genet..

[13] Tassone F., Iong K.P., Tong T.H., Lo J., Gane L.W., Berry-Kravis E., Nguyen D., Mu L.Y., Laffin J., Bailey D.B. (2012). FMR1 CGG allele size and prevalence ascertained through newborn screening in the United States. Genome Med..

[14] Lozano R., Rosero C.A., Hagerman R.J. (2014). Fragile X spectrum disorders. Intractable Rare Dis. Res..

[15] Jacquemont S., Hagerman R.J., Hagerman P.J., Leehey M.A. (2007). Fragile-X syndrome and fragile X-associated tremor/ataxia syndrome: Two faces of FMR1. Lancet.

[16] Murray A., Schoemaker M.J., Bennett C.E., Ennis S., Macpherson J.N., Jones M., Morris D.H., Orr N., Ashworth A., Jacobs P.A. (2013). Population-based estimates of the prevalence of FMR1 expansion mutations in women with early menopause and primary ovarian insufficiency. Genet. Med..

[17] Allen R.C., Zoghbi H.Y., Moseley A.B., Rosenblatt H.M., Belmont J.W. (1992). Methylation of HpaII and HhaI sites near the polymorphic CAG repeat in the human androgen-receptor gene correlates with X chromosome inactivation. Am. J. Hum. Genet..

[18] Lau A.W., Brown C.J., Penaherrera M., Langlois S., Kalousek D.K., Robinson W.P. (1997). Skewed X-chromosome inactivation is common in fetuses or newborns associated with confined placental mosaicism. Am. J. Hum. Genet..

[19] Thurman A.J., McDuffie A., Hagerman R., Abbeduto L. (2014). Psychiatric symptoms in boys with fragile X syndrome: A comparison with nonsyndromic autism spectrum disorder. Res. Dev. Disabil..

[20] Tassone F., Choudhary N.S., Tassone F., Durbin-Johnson B., Hansen R., Hertz-Picciotto I., Pessah I. (2013). Identification of expanded alleles of the FMR1 Gene in the Childhood Autism Risks from Genes and Environment (CHARGE) study. J. Autism Dev. Disord..

[21] Mazurczak T., Bocian E., Milewski M., Obersztyn E., Stanczak H., Bal J., Szamotulska K., Karwacki M.W. (1996). Frequency of Fra X syndrome among institutionalized mentally retarded males in Poland. Am. J. Med. Genet..

[22] Esposito G., Ruggiero R., Savarese G., Savarese M., Tremolaterra M.R., Salvatore F., Carsana A. (2013). A 15-year case-mix experience for fragile X syndrome molecular diagnosis and comparison between conventional and alternative techniques leading to a novel diagnostic procedure. Clin. Chim. Acta.

[23] Loesch D.Z., Huggins R.M., Bui Q.M., Taylor A.K., Hagerman R.J. (2003). Relationship of deficits of FMR1 gene specific protein with physical phenotype of fragile X males and females in pedigrees: A new perspective. Am. J. Med. Genet. A.

[24] Han X.D., Powell B.R., Phalin J.L., Chehab F.F. (2006). Mosaicism for a full mutation, premutation, and deletion of the CGG repeats results in 22% FMRP and elevated FMR1 mRNA levels in a high-functioning fragile X male. Am. J. Med. Genet..

[25] The Human Gene Mutataion Database. http://www.hgmd.org/.

[26] Pretto D.I., Mendoza-Morales G., Lo J., Cao R., Hadd A., Latham G.J., Durbin-Johnson B., Hagerman R., Tassone F. (2014). CGG allele size somatic mosaicism and methylation in FMR1 premutation alleles. J. Med. Genet..

[27] Heine-Suñer D., Torres-Juan L., Morla M., Busquets X., Barcelo F., Pico G., Bonilla L., Govea N., Bernues M., Rosell J. (2003). Fragile-X syndrome and skewed X-chromosome inactivation within a family: A female member with complete inactivation of the functional X chromosome. Am. J. Med. Genet. A.

[28] Godler D.E., Inaba Y., Shi E.Z., Skinner C., Bui Q.M., Francis D., Amor D.J., Hopper J.L., Loesch D.Z., Hagerman R.J. (2013). Relationships between age and epi-genotype of the FMR1 exon 1/intron 1 boundary are consistent with non-random X-chromosome inactivation in FM individuals, with the selection for the unmethylated state being most significant between birth and puberty. Hum. Mol. Genet..

[29] Ferreira S.I., Pires L.M., Ferrro J., Sa J., Serra A., Careira M. (2013). Mosaicism for FMR1 gene full mutation and intermediate allele in a female foetus: A postzygotic retraction event. Gene.

[30] Willemsen R., Bontekoe C.J., Severijnen L.A., Oostra B.A. (2002). Timing of the absence of FMR1 expression in full mutation chorionic villi. Hum. Genet..

[31] Wittenberger M.D., Hagerman R.J., Sherman S.L., McConkie-Rosell A., Welt C.K., Rebar R.W., Corrigan E.C., Simpson J.L., Nelson L.M. (2007). The FMR1 premutation and reproduction. Fertil. Steril..

[32] Jacquemont S., Hagerman R.J., Leehey M., Grigsby J., Zhang L., Brunberg J.A., Greco C., Des Portes V., Jardini T., Levine R. (2003). Fragile X Premutation Tremor/Ataxia Syndrome: Molecular, Clinical, and Neuroimaging Correlates. Am. J. Hum. Genet..

[33] Sherman S., Pletcher B.A., Driscoll D.A. (2005). Fragile X syndrome: Diagnostic and carrier testing. Genet. Med..

[34] Rajkiewicz M., Szlendak-Sauer K., Sulek A., Gawlik-Zawislak S., Krysa W., Radowicki S., Zaremba J. (2011). A molecular and cytogenetic investigation of FMR1 gene premutations in Polish patients with primary ovarian insufficiency. Eur. J. Obstet. Gynecol. Reprod. Biol..

[35] Pastore L.M., Johnson J. (2014). The FMR1 gene, infertility, and reproductive decision-making: A review. Front. Genet..

[36] Jacquemont S., Hagerman R.J., Leehey M.A., Hall D.A., Levine R.A., Brunberg J.A., Zhang L., Jardini T., Gane L.W., Harris S.W. (2004). Penetrance of the fragile X-associated tremor/ataxia syndrome in a premutation carrier population. J. Am. Med. Assoc..

[37] Jacquemont S., Leedey M.A., Hagerman R.J., Beckett L.A., Hagerman P.J. (2006). Size bias of fragile X premutation alleles in late-onset movement disorders. J. Med. Genet..

[38] Apartis E., Blancher A., Meissner W.G., Guyant-Maréchal L., Maltête D., de Broucker T., Legrand A.P., Bouzenada H., Thanh H.T., Sallansonnet-Froment M. (2012). FXTAS: New insights and the need for revised diagnostic criteria. Neurology.

[39] Adams S.A., Steenblock K.J., Thibodeau S.N., Lindor N.M. (2008). Premutations in the FMR1 gene are uncommon in men undergoing genetic testing for spinocerebellar ataxia. J. Neurogenet..

[40] Rajkiewicz M., Sułek-Piatkowska A., Krysa W., Zdzienicka E., Szirkowiec W., Zaremba J. (2008). Screening for premutation in the FMR1 gene in male patients suspected of spinocerebellar ataxia. Neurol. Neurochir. Pol..

[41] Filipovic-Sadic S., Sah S., Chen L., Krosting J., Sekinger E., Zhang W., Hagerman P.J., Stenzel T.T., Hadd A.G., Latham G.J. (2010). A novel FMR1 PCR method for the routine detection of low abundance expanded alleles and full mutations in fragile X syndrome. Clin. Chem..

[42] Hagerman R., Hagerman P. (2013). Advances in clinical and molecular understanding of the FMR1 premutation and fragile X-associated tremor/ataxia syndrome. Lancet Neurol..

[43] Latham G.J., Coppinger J., Hadd A.G., Nolin S.L. (2014). The role of AGG interruptions in fragile X repeat expansions: A twenty-year perspective. Front. Genet..

